# Interface design recommendations for computerised clinical audit and feedback: Hybrid usability evidence from a research-led system

**DOI:** 10.1016/j.ijmedinf.2016.07.010

**Published:** 2016-10

**Authors:** Benjamin Brown, Panos Balatsoukas, Richard Williams, Matthew Sperrin, Iain Buchan

**Affiliations:** Health eResearch Centre, Farr Institute of Health Informatics Research, Centre for Health Informatics, University of Manchester, Manchester, UK

**Keywords:** Clinical audit, Medical audit, Clinical quality management, Clinical quality improvement, Clinical governance, User interface design, Hybrid usability inspection, Usability studies, Clinical decision support systems

## Abstract

**Background:**

Audit and Feedback (A&F) is a widely used quality improvement technique that measures clinicians’ clinical performance and reports it back to them. Computerised A&F (e-A&F) system interfaces may consist of four key components: (1) Summaries of clinical performance; (2) Patient lists; (3) Patient-level data; (4) Recommended actions. There is a lack of evidence regarding how to best design e-A&F interfaces; establishing such evidence is key to maximising usability, and in turn improving patient safety.

**Aim:**

To evaluate the usability of a novel theoretically-informed and research-led e-A&F system for primary care (the Performance Improvement plaN GeneratoR: PINGR).

**Objectives:**

(1) Describe PINGR’s design, rationale and theoretical basis; (2) Identify usability issues with PINGR; (3) Understand how these issues may interfere with the cognitive goals of end-users; (4) Translate the issues into recommendations for the user-centred design of e-A&F systems.

**Methods:**

Eight experienced health system evaluators performed a usability inspection using an innovative hybrid approach consisting of five stages: (1) Development of representative user tasks, Goals, and Actions; (2) Combining Heuristic Evaluation and Cognitive Walkthrough methods into a single protocol to identify usability issues; (3) Consolidation of issues; (4) Severity rating of consolidated issues; (5) Analysis of issues according to usability heuristics, interface components, and Goal-Action structure.

**Results:**

A final list of 47 issues were categorised into 8 heuristic themes. The most error-prone heuristics were ‘Consistency and standards’ (13 usability issues; 28% of the total) and ‘Match between system and real world’ (n = 10, 21%). The recommended actions component of the PINGR interface had the most usability issues (n = 21, 45%), followed by patient-level data (n = 5, 11%), patient lists (n = 4, 9%), and summaries of clinical performance (n = 4, 9%). The most error-prone Actions across all user Goals were: (1) Patient selection from a list; (2) Data identification from a figure (both population-level and patient-level); (3) Disagreement with a system recommendation.

**Conclusions:**

By contextualising our findings within the wider literature on health information system usability, we provide recommendations for the design of e-A&F system interfaces relating to their four key components, in addition to how they may be integrated within a system.

## Highlights

• Presents a novel theoretically-informed computerised audit and feedback (e-A&F) system.
• Identifies usability issues by employing an innovative hybrid inspection approach.
• Translates findings into recommendations for the user-centred design of e-A&F systems.

## 1 Introduction

Audit and feedback (A&F) is an established and widely used technique in quality improvement, employed in health care systems across the world. It consists of measuring a clinician or health care team’s clinical performance over a specified period of time (audit), and reporting it to them (feedback), with the intention of raising awareness and helping them take corrective action [1]. Audit data are obtained from medical records, computerised databases, or observations from patients, and feedback may include recommendations for improvement action [2].

In A&F, clinical performance is measured by adherence to recommended clinical practices (e.g. patients with hypertension receiving regular blood pressure measurements) or the occurrence of particular patient outcomes (e.g. acceptable blood pressure control) [1], [2]. A&F relates to care provided to multiple rather than individual patients, and is used to inform improvements at an individual, team, and service level [3], [4]. Feedback relating primarily to individual patients, particularly intended for use at the point of care, does not count as A&F, and is classified as a different intervention such as clinical decision support (CDS) [1], [2].

A&F is traditionally undertaken using paper medical records, which is laborious and time-intensive. However, widespread use of electronic health records (EHRs) has spawned a variety of computerised A&F systems (e-A&F). These systems usually feed audit results back to provider employees via interactive interfaces such as intranet browser-based portals (e.g. [5]) or desktop applications (e.g. [6]). Users of e-A&F systems are generally clinicians whose performance is being assessed, though may also include managers or administrators [7]. e-A&F systems are distinct from systems where an audit is generated using a computerised infrastructure but feedback is provided on paper, verbally or via a static computerised form such as a screensaver or electronic document (e.g. [8]). Often e-A&F systems are not explicitly termed ‘audit and feedback’, and instead may be called ‘dashboards’, ‘scorecards’, ‘business intelligence’, ‘visualisation tools’ or ‘benchmarking tools’ amongst other names [9]. Conversely, many systems with these names may also *not* be A&F: for example, many dashboards only provide information regarding individual patients (e.g. clinical dashboards [10]) or may focus on multiple patients but are intended for use solely at the point of care (e.g. [11]); and business intelligence or information visualisation tools may focus primarily on non-clinical performance data such as costs, patient waiting times, or disease epidemiology surveillance (e.g. [12]).

Despite their prevalence, there has been relatively little research into the requirements for designing usable interfaces for e-A&F systems. Prior work has largely focused on the effectiveness of e-A&F systems for improving patient care (e.g. [13]) or their levels of adoption (e.g. [14]). Some studies have explored factors related to their acceptance and use (e.g. [15]), however, we are aware of only one study that has explicitly focused on usability [16]. Consequently little is known about how best to design e-A&F interfaces.

Ongoing work by our group has identified four key components of e-A&F system interfaces [17]: (1) Summaries of clinical performance; (2) Patient lists; (3) Patient-level data; and (4) Recommended actions. All e-A&F interventions have some combination of these elements; indeed, to qualify as A&F the system must have at least a summary of clinical performance or provide patient lists [1], [2], [3], [4]. However, we are unaware of a system reported in the literature that incorporates all four components. Below, we discuss each interface component, and what is currently known about their usability.

### 1.1 Summaries of clinical performance

A&F interventions generally summarise clinical performance using quantitative measures variably termed ‘quality indicators’, ‘performance measures’ or similar. They usually report the proportions or absolute numbers of patients who have (or have not) received a recommended clinical practice, or experienced a particular outcome [18]. These metrics are the core component of A&F, and are commonly presented either as tables (e.g. [19]), bar plots (e.g. [20]), pie charts (e.g. [21]), or line graphs (e.g. [15]). Sometimes colour coding (e.g. [22]) or comparison with peers (e.g. [23]) are used to highlight progress towards desirable levels of performance (termed targets or goals). In terms of usability, the use of line graphs to monitor trends in performance in an e-A&F system have been found to be useful, in addition to the ability to interactively explore aggregated patient data, and compare performance between departments within an organisation [16]. However, it is unclear how these functions should be optimally designed, or integrated with other formats of data presentation.

### 1.2 Patient lists

Some e-A&F systems provide lists of patients who have (e.g. [24]) or have not (e.g. [15]) received the recommended clinical practice, or experienced the particular outcome of interest. This is generally supplemental to the summary of clinical performance (e.g. [20]), though occasionally may act as its proxy (e.g. [19]). The intention in providing patient lists is that they can be used to further investigate the care of individual patients and take corrective action where necessary [25]. Patient lists have been identified as a key driver of success in some non-computerised A&F interventions [26], and their absence as a reason for failure [27]. They may simply contain patient names or identifiers, or additional summary data such as demographics or physiological measurements (e.g. [20]). We are unaware of any published studies of e-A&F interventions that have assessed the usability of patient lists, so evidence regarding their optimal design is lacking. For example, it is unclear how they should be integrated with the summary of clinical performance, or how (and whether) they should include patient-specific summary data as a means of improving information processing and cognitive load during interpretation tasks.

### 1.3 Patient-level data

e-A&F systems may occasionally further supplement patient lists with more detailed information about each patient (e.g. historic glycated haemoglobin readings for diabetic control [28]). Access to these data, whether within the e-A&F system itself or the EHR, is key so that individual patients’ care can be reviewed, and action taken where necessary [27]. In e-A&F systems, such information may be presented in tables (e.g. [15]) or graphically (e.g. [16]). From a usability point of view, integrating patient-level with population-level data in an e-A&F system has been demonstrated as desirable to users, and that functionality should support information visualisation over predefined time periods in addition to interactive exploration [16]. Similarly, a usability evaluation of a primary care epidemiological visualisation tool found that providing these data within the system was advantageous as clinicians may not have time to check each patient’s EHR [29]. However, it is unknown how best to present such detailed patient-level data within an e-A&F system, or how much data to present without overwhelming the user and increasing cognitive load during task performance [30].

### 1.4 Recommended actions

The definition of A&F states that recommended actions for improvement may accompany clinical performance feedback [2]. There is both theoretical [31] and empirical evidence [1] that providing recommended actions increases the effectiveness of A&F. Often A&F recipients do not have the time, capacity or skills to interpret feedback and formulate what improvement action is necessary [27], so providing recommendations increases the likelihood that action is taken [31]. User-needs assessments for e-A&F systems often find that recommended actions are desirable [23]), and some systems provide links to educational materials such as best practice guidelines (e.g. [28]) or templates for users to formulate their own action plans (e.g. [32]), however we are only aware of one e-A&F system in which improvement actions are actually recommended to users (the LPZ Dashboard [23]). The recommendations in this system are generic and target organisational changes only, which the user derives themselves using a decision tree [23]. The usability of this system was not evaluated, so it is unclear how best to present recommended actions within an e-A&F system.

In addition to the knowledge gaps regarding each of the four interface components described above, there is also little insight into how they should be effectively integrated in a single e-A&F system in a manner that aids information processing, minimises technology-induced errors, and reduces cognitive load during interaction. It is therefore important to investigate the usability of e-A&F systems in more depth to produce evidence that can guide their design. Developing health information systems without regard for user interaction can reduce their effectiveness, with adverse consequences for patient safety and care quality [33], [34]. This is particularly important for e-A&F systems, where the none-use or misuse of clinical performance data can lead to suboptimal care on a large scale with important adverse implications for patient outcomes and cost (e.g. [35]). Conversely, effective use of A&F has the potential to vastly improve care quality: the latest Cochrane review of A&F found it can increase desired care processes by up to 70% [1], which if multiplied across large populations can lead to major gains. However if used ineffectively, A&F can decrease desired care processes up to 9% [1]. Whether A&F is effective or ineffective is partly determined by how clinical performance feedback data is presented to users [1].

## 2 Aim and objectives

The aim of this paper is to address the gaps in the literature identified above by evaluating the usability of an e-A&F system for primary care (the Performance Improvement plaN GeneratoR; PINGR). To the best of our knowledge, PINGR is the first reported system to comprise all four interface components found in e-A&F applications (summaries of clinical performance, patient lists, patient-level data, and recommended actions). Further originality of PINGR relates to its design being informed by existing usability evidence and relevant behaviour change theory (we are aware of only two reported e-A&F systems that explicitly incorporated existing usability guidelines and theory in their design [15], [36]). To evaluate the PINGR system, we adopted an iterative approach to system design involving multiple evaluation cycles at different stages of the development process [37]. This paper reports on the findings of the usability inspection study during the first part of our evaluation cycle. The specific objectives of this paper are to:

1. Describe PINGR’s interface design, rationale and theoretical basis;
2. Identify usability issues with PINGR in relation to its four interface components;
3. Understand how these issues may interfere with the cognitive goals of end-users (and therefore the integration of the interface components);
4. Translate these issues into recommendations for the user-centred design of e-A&F systems in general.

The paper is structured as follows: in the next section we present an overview of the PINGR system, and discuss its design and theoretical justification by drawing on relevant design guidelines, usability research and theory (Objective 1). The following two sections then report the methodology and results of the usability inspection study (Objectives 2 and 3). The final section presents a discussion of the results and design recommendations (Objective 4).

## 3 The Performance Improvement plaN GeneratoR (PINGR)

PINGR is a web-based e-A&F tool designed to help UK primary care clinicians improve the quality of care they provide to patients. The version presented in this paper focuses on use cases of hypertension and asthma. PINGR was conceived and designed by author BB (a UK primary care physician and health informatics researcher), and was built by both authors RW (a software engineer) and BB. It is intended for use by clinicians outside patient consultations to assess the care provided by a primary care practice to its patient population, and to inform subsequent improvement actions at an individual, team, and service level. It analyses EHR data in the form of Read v2 codes, though has the capability to handle any type of structured data. These data are processed in a SQL Server database optimised for query execution. PINGR’s interface is built with HTML, JavaScript and CSS, using libraries including Bootstrap (http://getbootstrap.com/), C3.js (http://c3js.org/), jQuery, and Mustache.js (http://mustache.github.io/). Given the paucity of research into e-A&F system usability, its design primarily draws on literature regarding user needs and theory for A&F interventions identified in ongoing work by our group [17], in addition to design guidelines for other types of health information system. These design guidelines were selected based on their similarity and relevance to each of PINGR’s four interface components (summaries of clinical performance, patient lists, patient-level data, and recommended actions). For example: recommendations for displaying quantitative information (e.g. [38]) were used to inform the design of summaries of clinical performance because they contain quantitative quality indicators; EHR design guidelines (e.g. [39]) were used for the design of patient lists and patient-level information because they are common elements of EHRs; and CDS system design guidelines (e.g. [40]) were used to inform the design of recommended actions because they often suggest actions for users [41]. The remainder of this section describes the design and rationale of PINGR’s interface components.

### 3.1 Summaries of clinical performance

**Fig. 1:**
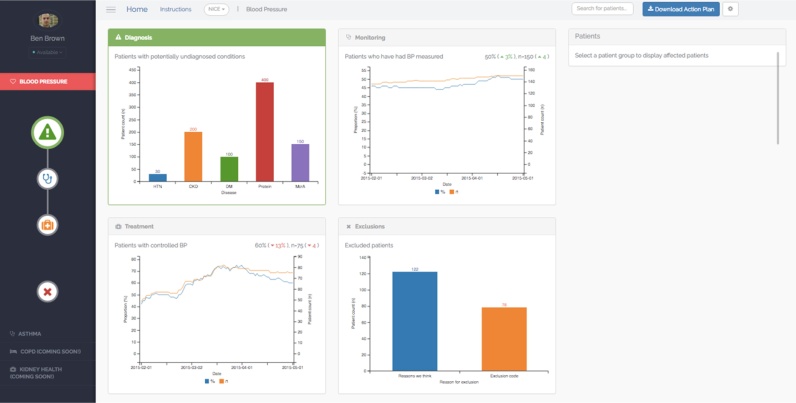
Overview page (example is hypertension).

**Fig. 2:**
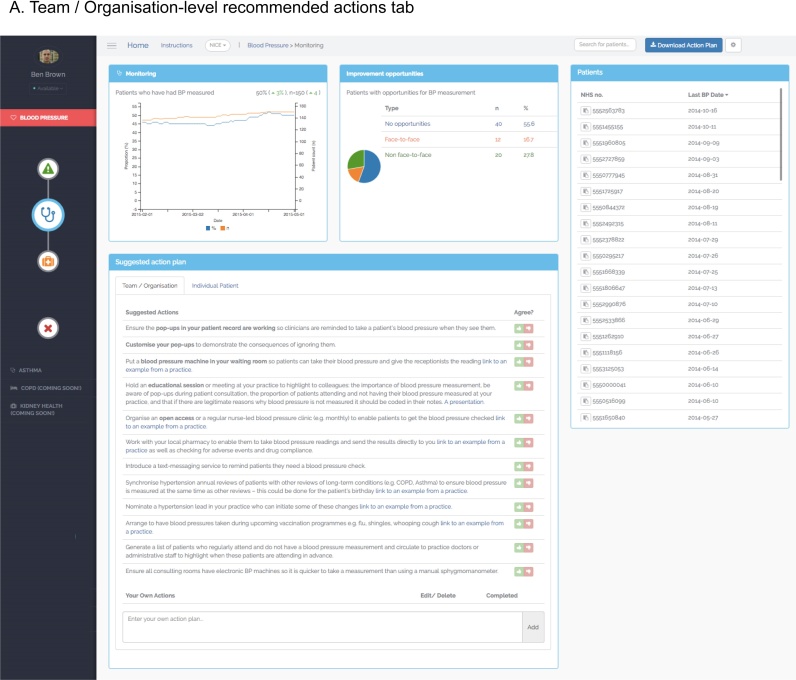

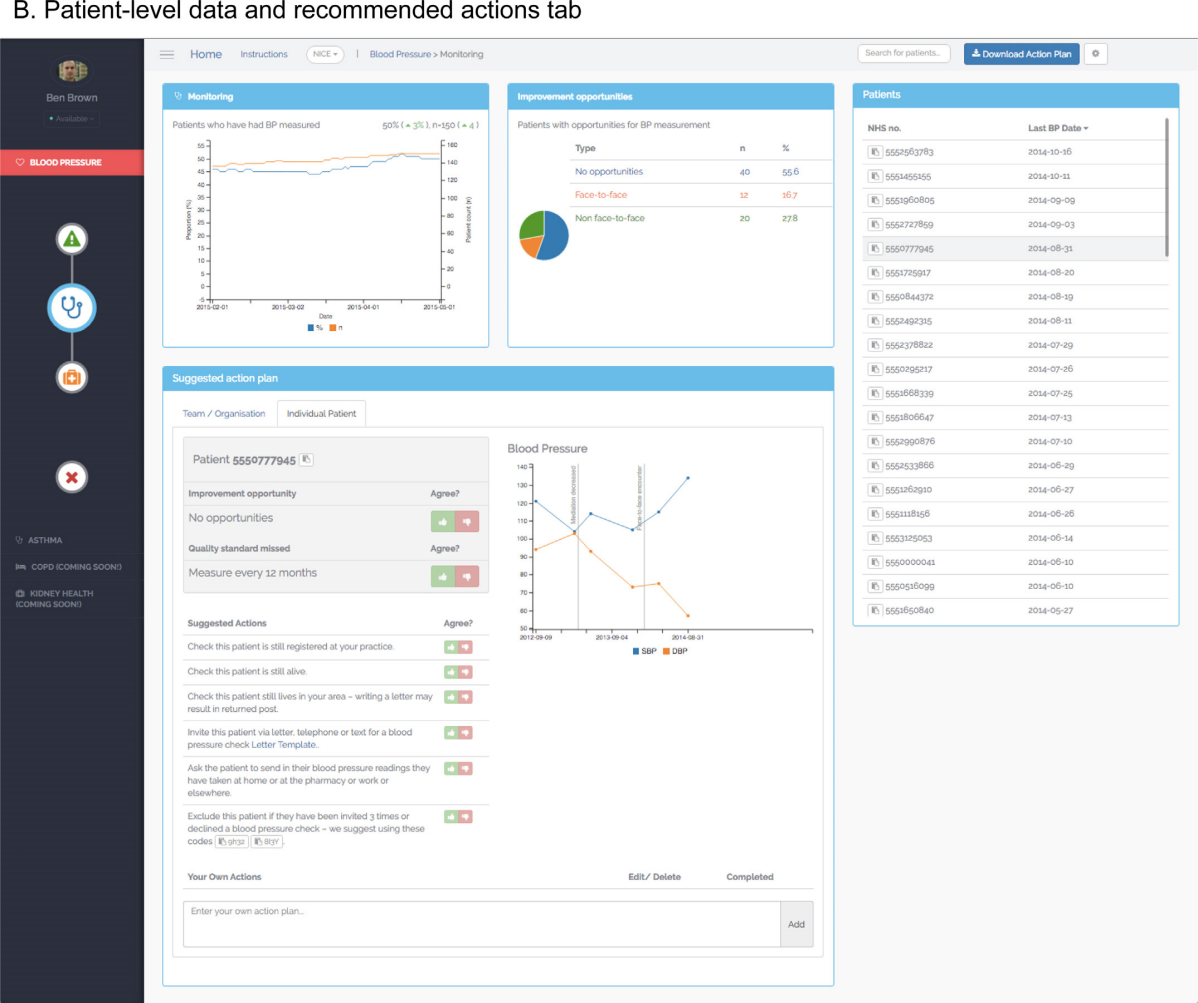
Preview page (example is hypertension monitoring). 2A. Team/Organisation-level recommended actions tab. 2B. Patient-level data and recommended actions tab.

Clinical performance summaries for each clinical area within PINGR are organised as separate modules, accessed from an icon-based menu on the left side of the interface (Fig. 1). A module-oriented design was employed to enhance information processing as demonstrated in clinical guidelines [42] and general web design [43]. Within each clinical module, there are 2 pages: (1) an Overview page (Fig. 1), and (2) a Preview page (zoom and filter; Fig. 2a and b). Overview and preview has been widely used in the design of applications to support visual information-seeking tasks [44]. After selecting a module from the menu, the Overview page is displayed which presents the primary care practice’s clinical performance as quality indicators. These quality indicators (described in further detail below) convey proportions and absolute numbers of patients who have received a recommended clinical practice, or experienced a particular outcome. Using both relative and absolute measures of performance avoids potentially misleading effects of providing isolated measures of relative performance [45]. To create a generic template for all clinical conditions, and consistency of interface design as recommended in EHR [39] and CDS system [40] usability guidelines, PINGR organises quality indicators into four common areas along a clinical condition pathway: diagnosis, monitoring, treatment, and exclusions. To illustrate, the hypertension area of the system displays the following elements: patients with diagnosed hypertension (and other relevant conditions such as chronic kidney disease or diabetes) based on their prior recorded measurements (diagnosis); hypertensive patients who have had their blood pressure measured in the preceding year (monitoring); hypertensive patients whose latest blood pressure measurement is within their recommended personalised target (control); and hypertensive patients who have been excluded from quality standards, such as those with a terminal illness (exclusions). In accordance with data visualisation design principles [38], and to reduce short-term memory load [46], the four quality indicators are presented as separate panels on a single screen to provide the user with an overview of the practice’s clinical performance in that disease area. Quality indicators are displayed as line graphs for trend visualisation (i.e. in monitoring and control indicators), and bar plots for processing of one-off data points where it was anticipated to be most clinically informative (i.e. diagnosis and exclusions), which is supported by feedback intervention theory [31], cognitive fit theory [47], and evidence from a usability study [16]. Each graph is supplemented with labels to indicate the current level of clinical performance, interactive tool-tips to detail historical performance, and icons to highlight the change in performance from the previous month [38].

Users can request further information regarding their clinical performance by clicking on the quality indicator graphs thereby accessing the Preview page (Fig. 2a and b). The Preview page is organised with the quality indicator graph in the top left hand corner, with the remaining interface elements (discussed in detail below) arranged with patient lists on the right, and patient-level data and recommended actions at the bottom. This layout mirrors the anticipated reading pattern [48] and workflow that users would follow: reviewing their summary of performance, then list of patients requiring action, followed by detailed patient-level information, and recommended improvement actions. Displaying all 4 interface components on one page was also intended to reduce cognitive load and improve task completion by supporting recognition rather than recall of available user options [46].

### 3.2 Patient lists

Patient lists are populated with patients who have not achieved the clinical standard or desired outcome in the quality indicator to help users take corrective action where appropriate. The lists included patients’ unique identification number, which can be cross-referenced with an EHR system using a ‘copy’ icon to prevent errors [39]. An additional column displays clinical data felt most relevant to the quality indicator (e.g. latest blood pressure reading for the ‘control’ quality indicator), which can be used to order the list and prioritise patients for action. Ordering and prioritisation patient lists has been indicated as valuable in non-computerised A&F interventions [27], and is consistent with design guidelines for EHRs [49]. The current version of PINGR only provides one column of patient attributes based on empirical evidence that displaying multiple clinical variables can adversely affect the usability of primary care epidemiological visualisation tools [29]. The lists can be filtered by selecting sections of the ‘improvement opportunity’ graph (see recommended actions section below), acting as an interactive visual query mechanism as recommended by usability research into e-A&F systems [16], EHRs [49] and quantitative data display in general [44].

### 3.3 Patient-level data

When a specific patient is selected from a list on the Preview page, the bottom panel displays an ‘individual patient’ tab (Fig. 2B), which can also be accessed by entering a patient’s unique identifier into the search bar located in a menu at the top of the screen. Here detailed patient-level data relevant to the quality indicator is presented, in addition to patient-level recommended actions (which are discussed in detail below). In the hypertension module this information relates to patients’ blood pressure measurements, whereas in the asthma module it relates to their peak expiratory flow rate. Based on previous usability research into e-A&F systems [16], these data are presented as line graphs. Interactive tool-tips provide detail on historical data as recommended in EHR usability guidelines [50]. Further information on relevant non-physiological events are also presented on the graphs as vertical lines such as when a patient had an encounter with the practice, or experienced a change in medication.

### 3.4 Recommended actions

PINGR provides recommended actions in the bottom panel of the Preview page (Fig. 2). In contrast to the LPZ Dashboard system [23], these recommendations address both the organisation (i.e. the primary care practice) *and* individual patients, are specific to users’ clinical performance (rather than generic), and are provided automatically (rather than on-demand). These design choices were based on existing evidence and theory: providing two types of recommended action is consistent with health care quality improvement theory [51], whereas providing tailored recommended actions in a user’s workflow is recommended in CDS system design [40], [52]. In this sense, PINGR can be viewed as a cross-fertilisation of traditional A&F and CDS systems producing ‘care-system-level decision support’, which we have previously argued could lead to greater effectiveness of both types of system [41].

Recommended actions are derived through further analysis of contextual data of patients who have not achieved the quality standards, which is supported by CDS system design guidelines [53]. These patients are subsequently grouped into ‘improvement opportunity’ categories that infer potential *reasons* why patients have not achieved the quality standards or outcomes of interest, and are associated with a specific set of potential *solutions* in the form of recommended actions (both at the organisational and patient levels). The improvement opportunity categories and bank of recommended actions are generated from clinical guidelines, research literature (e.g. [54]), and empirical analysis of medical records [55]. To illustrate: in the hypertension monitoring quality indicator, improvement opportunity categories relate to patients’ contact with the primary care practice: either face-to-face, non face-to-face (e.g. over the phone), or no contact [55]. An algorithm analyses EHR data from each hypertensive patient who has not met the quality standard, makes inferences regarding the type of contact each patient had with the practice [55], and provides relevant recommended actions to help these patients attain the quality standard.

The proportions of patients in each improvement opportunity category are displayed in a panel to the right of the quality indicator graph as a pie chart (Fig. 2a and b), which act as the visual query mechanism to filter the patient list described above. Clicking on a section of the pie chart filters the list to display each patient in that improvement opportunity category. The intention was that this would facilitate user action by grouping patients associated with similar improvement tasks, thus minimising cognitive load [30]. The recommended actions are automatically displayed in a table, where users can also add their own actions as free text (Fig. 2a and b). Users can agree or disagree with them by clicking a ‘thumbs up’ or ‘thumbs down’ icon respectively (Fig. 2a). If a user agrees with a recommended action, it turns green and is saved to their personal bank of actions in the home page in accordance with CDS system usability design [56]. Users can indicate when a task has been completed using a check box, and can download their list of agreed actions as a document to print or share. If a user disagrees with an action, a dialogue box captures the reasons for this using fixed responses or free-text as recommended in the design of EHRs and CDS systems [39], [52], [53]. Framing recommended actions as advice rather than commands is in accordance with design guidelines for CDS systems [40], and asking for reasons for override has been shown to improve their effectiveness [57].

## 4 Materials and methods

We evaluated usability issues associated with PINGR using a hybrid usability inspection method, which combined Heuristic Evaluation (HE) and Cognitive Walkthrough (CW). Usability inspection methods involve experienced evaluators assessing a system to identify issues that could potentially hinder user interaction with the software. They are recommended as a cost-efficient initial step in usability evaluation as they can identify a wide range of issues without the need for real end-users (in this case, primary care clinicians) or significant resources [58]. At this stage of our iterative evaluation process, the involvement of experienced evaluators was necessary to identify and correct critical usability issues according to established usability guidelines. In accordance with accepted usability engineering methodology, real end-users will be involved in future evaluation rounds of PINGR to capture any issues that may have been overlooked [34].

HEs and CWs are often recommended to be carried out separately on a system [34], which has both advantages and disadvantages. HEs assess interfaces against a set of well-established design guidelines known to play an important role in user experience, and do not restrict the evaluator to interact with the interface in a specific way, thereby maximising usability issue discovery [58]. This is important for e-A&F systems in general where there is a lack of usability knowledge, and for the PINGR system in particular, which has not been previously evaluated. However, among other limitations (e.g. [59]), HEs do not adequately explore how issues arise during user interaction with a system beyond its static interface features, nor how they relate to the user’s cognitive needs [60]. This is particularly important in health IT systems, such as PINGR, with dynamic user interfaces that require complex interactions to achieve user goals [61]. This limitation can be addressed by the CW method, though traditionally CWs do not take advantage of accepted usability heuristics, which may limit their ability to identify potential issues [62]. Independent HEs and CWs often discover different usability issues in the same system [63], making it cumbersome to combine their relative advantages if used separately. Therefore as the complexity of health information systems progress, there is a need to harness the combined benefits of HEs and CWs into a single hybrid usability inspection technique; though as yet, little progress has been made [61].

### 4.1 Participants and setting

We recruited a convenience sample of health information system evaluators from the Centre for Health Informatics, University of Manchester. Eligible participants were qualified software developers or evaluators with more than five years’ experience in health information system design and development. Using three to five usability experts is recommended in HEs as a balance between costs and benefits, and is expected to detect around 75% of usability issues in a system [64], [65]. However, given our objective was to identify as many unique usability issues as possible, we invited eight potential evaluators, all of whom accepted. None of the evaluators had previously used PINGR, though all had experience of using similar systems such as non-clinical dashboards, and epidemiological surveillance tools. All stages of the evaluation took place at the University of Manchester where evaluators accessed the PINGR application via the Google Chrome web browser on a 17-inch computer screen. To preserve patient privacy, we used simulated data for the purposes of the usability inspection.

### 4.2 Hybrid inspection method

**Fig. 3:**
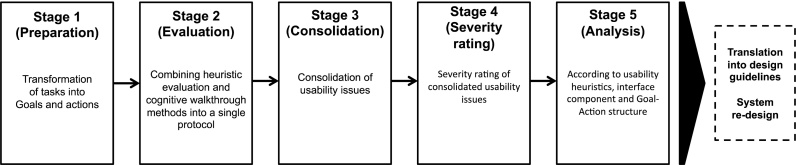
Overview of hybrid usability inspection methodology.

Our hybrid method incorporated elements of both HE and CW, adapting the approach advocated by Kushniruk et al. [61]. It comprised five stages (Fig. 3): (1) Development of representative user tasks and their transformation into goals and actions; (2) Combining HE and CW methods into a single protocol to identify usability issues regarding the PINGR application; (3) Consolidation of usability issues identified by evaluators in stage 2; (4) Severity rating of consolidated usability issues; (5) Analysis according to usability heuristics, interface component, and Goal-Action structure. These stages are described in further detail below.

#### 4.2.1 Stage 1: development of representative user tasks and their transformation into goals and actions

**Fig. 4:**
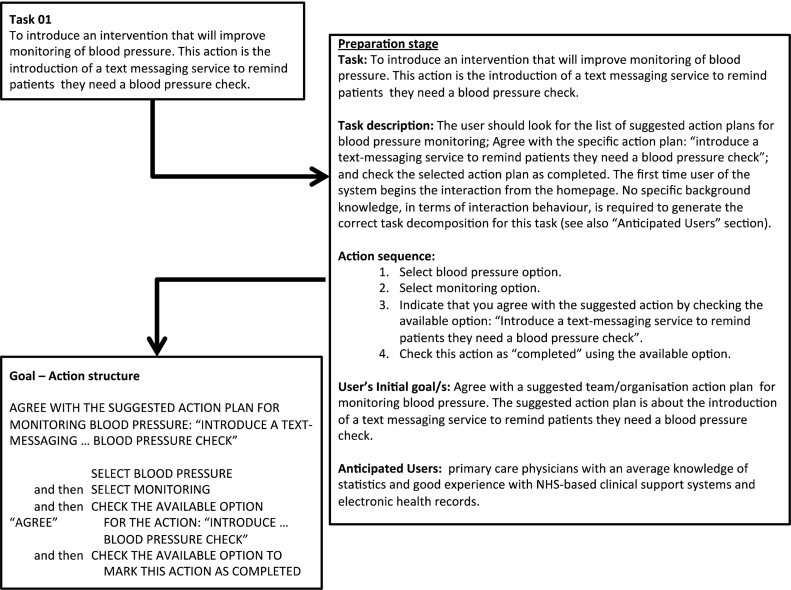
Demonstration of process for deriving Goal-Action structure from user tasks.

Initially we followed the typical procedure for a CW evaluation by describing tasks and their associated goals to be used in the evaluation (Fig. 4; Task description; User’s initial goal/s) [66]. Eight representative user tasks were selected, piloted and refined, to guide interaction with all components of the PINGR interface (Table 1). Each task was decomposed into up to 8 constituent actions, and their optimal sequence determined to minimise cognitive effort to achieve each task’s goal (Fig. 4; Action sequence) [66]. There were 44 actions in total across the 8 tasks. Characteristics and needs of intended users were also described for each task (Fig. 4; Anticipated users). This information was used to produce a Goal-Action structure document (Appendix A in Supplementary material) to contextualise each task for the interface evaluators in the next stage.

#### 4.2.2 Stage 2: combining HE and CW methods into a single protocol to identify usability issues

Evaluators worked independently rather than as a group in order to identify a larger and more diverse number of usability issues [67]. Each evaluator met individually with author BB face-to-face; they were introduced to the objectives and methods of the study, and the aims, high-level functionality, and rationale of the PINGR system using a standardised script. A demonstration of how to use PINGR was *not* provided in order to evaluate the learnability of the system [68]. As in standard CW protocol, each evaluator then investigated the interface following the tasks in the Goal-Action structure document (Appendix A in Supplementary material). For each Action the evaluator applied Nielsen’s 10 usability heuristics to identify usability issues according to its categories [69]: (1) Visibility of system status; (2) Match between system and the real world; (3) User control and freedom; (4) Consistency and standards; (5) Error prevention; (6) Recognition rather than recall; (7) Flexibility and efficiency of use; (8) Aesthetic and minimalist design; (9) Help users recognise, diagnose, and recover from errors; (10) Help and documentation. These generic heuristics were chosen due to a lack of specific heuristics for e-A&F systems. If a usability issue was identified, evaluators took screenshots and described it in detail in an electronic data collection form (Appendix B in Supplementary material). They also recorded the task and Goal-Action(s) in which it occurred, the heuristic category with which it was associated, and their rating of its severity on a 4-point scale [58] (Table 2). Both the heuristic categories and severity ratings were provided in an electronic document for reference. To make the process more constructive, evaluators also provided suggestions as to how each issue could be improved (if this was not obvious), and once they had completed the tasks listed up to three positive aspects of the system. Any missing data or unclear descriptions were clarified by BB who was present throughout the process. As in a standard HE, participants were encouraged to explore usability issues outside the specified goal-action structure to assess general aspects of PINGR's functionality and record them under the relevant task. Each participant took on average one hour to perform their evaluation, and in total identified 132 issues with a mean severity of 2.

#### 4.2.3 Stage 3: consolidation of usability issues

**Fig. 5:**
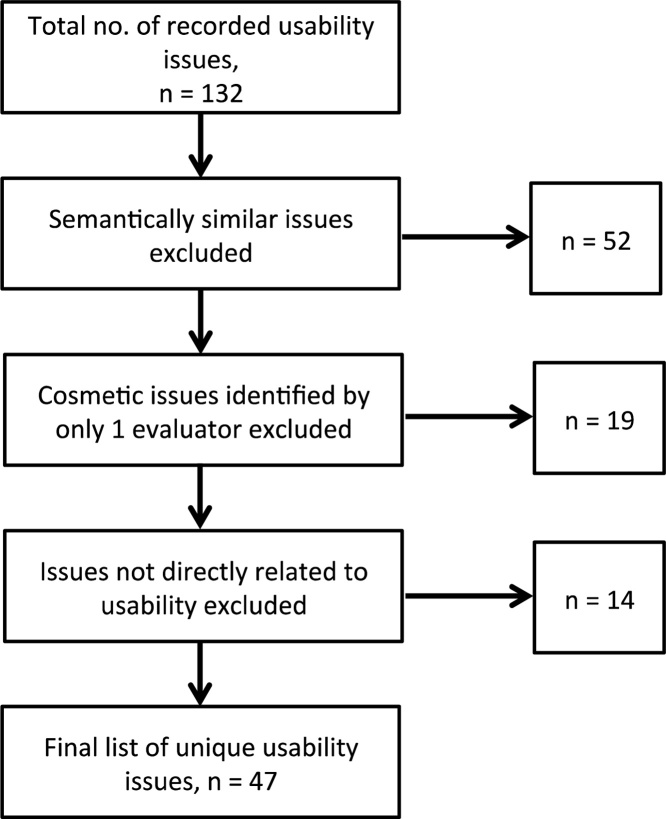
Flowchart of usability issue discovery and finalisation.

All usability issues collected from each evaluator in stage 2 were collated into a single document. Two authors (BB and PB) worked independently to consolidate the issues using an interpretivist approach by: (1) Integrating semantically similar issues into one issue; (2) Removing issues identified by only one evaluator, and rated as a ‘cosmetic issue only’ (Table 2) to reduce the occurrence of ‘false positive’ issues associated with traditional HEs [70]; (3) Removing issues not directly related to the usability of the application, such as suggestions for new system functionality; and (4) Assigning each consolidated issue to the most appropriate heuristic category, component of PINGR’s interface, and task Action(s) in which it arose. The final list was agreed through discussion, which consisted of 47 unique usability issues (Fig. 5). Out of these 47 issues, 24 (51%) had been identified by a single evaluator, of which 15 were rated as ‘minor’ (63%), 7 as ‘major’ (29%), and 2 as ‘usability catastrophes’ (8%; Table 2). This suggests our decision to use multiple evaluators working independently achieved our objective of identifying as many diverse usability issues as possible.

#### 4.2.4 Stage 4: severity rating of consolidated usability issues

Each evaluator who participated in Stage 2 was sent by e-mail a list of the finalised usability issues and asked to rate their severity using an electronic questionnaire (Appendix C in Supplementary material). The task number(s) and location in the Goal-Action structure with which each usability issue was associated was provided. The same severity rating scale was used from Stage 2, though to account for issues being identified by only one evaluator in Stage 2 an additional point was added: ‘I don’t agree that this is a usability issue at all’ (non-issues) [58]. Due to the gap of approximately one week between Stage 2 and Stage 4 that could have adversely affected participants’ recall of the issues, a hyperlink to PINGR was provided along with the original list of tasks used in stage 2 (Appendix A in Supplementary material). Participants were encouraged to remind themselves of usability issues they had previously identified, and familiarise themselves with issues that had been identified by others by navigating the system again using the Goal-Action structure.

#### 4.2.5 Stage 5: statistical analysis

We calculated the mean severity rating for each issue to the nearest integer to aid interpretation and prioritisation according to our scale (Table 2). Issues and positive comments were subsequently analysed thematically, and grouped according to interface component, and by their occurrence during user Goals and Actions. For stage 4, we measured inter-rater agreement (IRA), the extent to which evaluators assigned the same value for each item, and inter-rater reliability (IRR), the extent to which different evaluators consistently distinguished between different items on the severity scale [71]. We evaluated IRA by calculating simple proportions of agreement, and the Kendall coefficient of concordance adjusted for ties [71]. We evaluated IRR by calculating intra-class correlation coefficients (ICC) using a one-way model to estimate consistency of single ratings; Light’s weighted kappa; and Krippendorff’s alpha [71]. All measures of IRA and IRR used range between 0 and 1, with 1 signifying complete agreement or reliability. All analyses were performed using R [72], and the packages ‘irr’ [73] and ‘psy’ [74].

## 5 Results

**Fig. 6:**
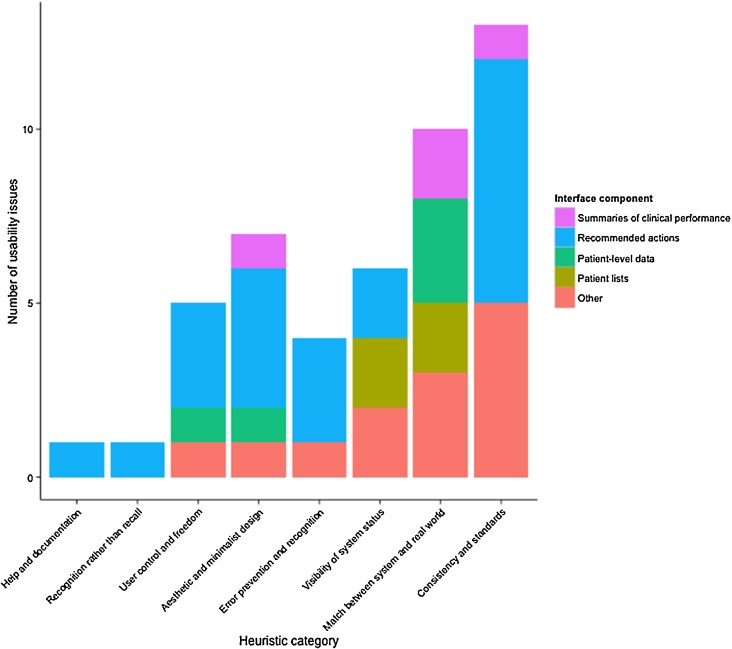
Stacked bar plot of usability issues per heuristic category versus interface components.

The final list of 47 issues were categorised into 8 heuristic themes (Fig. 6): ‘Flexibility and efficiency of use’ had no usability issues, and we combined the heuristics ‘Error prevention’ and ‘Help users recognise, diagnose, and recover from errors’ (‘Error prevention and recognition’) due to their issues’ conceptual similarity. The most error-prone heuristics were ‘Consistency and standards’ (13 usability issues; 28% of the total) and ‘Match between system and the real world’ (n = 10, 21%). The least violated heuristics were ‘Recognition rather than recall’ (n = 1, 2%), and ‘Help and documentation’ (n = 1, 2%). Analysis of mean severity ratings revealed 12 (26%) major usability issues, 26 (56%) minor issues, and 9 (19%) cosmetic issues; no usability catastrophes or non-issues were identified. Twenty-four positive comments were made about PINGR, 13 (54%) of which related to the system in general, praising its clean and visually appealing design, responsiveness, intuitive and simple layout, and use of contextual tool-tips. All eight evaluators did not agree on the exact severity of any issues, though within a tolerance of one point agreed on the severity of 8 (17%). Kendall’s coefficient of concordance was 0.44, indicating weak-moderate agreement [75], [76]. The ICC and Weighted Light’s Kappa statistic were both 0.33, whilst Krippendorff's alpha reliability coefficient was 0.04, indicating poor-fair reliability [77], [78].

### 5.1 Interface components

Recommended actions had the most usability issues (n = 21, 45%), followed by patient-level data (n = 5, 11%), patient lists (n = 4, 9%), and summaries of clinical performance (n = 4, 9%). The remaining 13 (28%) issues were associated with other non-unique aspects of the PINGR interface concerned with system navigation. The recommended actions received the most positive comments (n = 5, 21%), followed by the summaries of clinical performance (n = 4, 17%), and patient-level data (n = 2, 8%). Patient lists received no positive comments. Below we present these usability issues in detail organised by heuristic, and discuss positive comments. For brevity we only describe issues in detail with a mean severity rating of ‘minor’ or above.

#### 5.1.1 Summaries of clinical performance

Issues with summaries of clinical performance were categorised under the heuristics ‘Match between the system and the real world’ (n = 2), ‘Consistency and standards’ (n = 1), and ‘Aesthetic and minimalist design’ (n = 1). Under ‘Match between the system and the real world’, issues concerned the use of a cross icon to represent excluded patients as this is generally used to indicate an exit action, and that bar plot data points required clearer labelling. Under ‘Consistency and standards’, it was sometimes unclear what aspects of clinical performance the quality indicators were specifically measuring (rated as at least a ‘major’ usability issue by 4 out of 8 evaluators), whilst in ‘Aesthetic and minimalist design’ it was noted that plots did not re-size well with the internet browser window. Positive comments were made about the use of different colours to indicate the 4 pathways (diagnosis, monitoring, treatment, exclusions), and that although users were presented with a lot of information, it was generally felt to be easy to understand, particularly with the use of tool-tips to find historical performance data on line graphs.

#### 5.1.2 Patient lists

Issues with patient lists were categorised under the heuristics ‘Match between the system and the real world’ (n = 2) and ‘Visibility of system status’ (n = 2). Under ‘Match between the system and the real world’, issues concerned: a lack of clarity as to what the different lists referred (rated as at least a ‘major’ usability issue by 7 out of 8 evaluators); difficulty in browsing due to the lack of visible ordering options; and the need to use more useful parameters by which they could be ordered. Under ‘Visibility of system status’, issues concerned a lack of feedback when a new patient had been selected, or when a list had been filtered by interacting with the improvement opportunities graph (rated as at least a ‘major’ usability issue by 6 out of 8 evaluators).

#### 5.1.3 Patient-level data

Issues with patient-level data were categorised under ‘Match between the system and the real world’ (n = 3), ‘Aesthetic and minimalist design’ (n = 1), and ‘User control and freedom’ (n = 1). Under ‘Match between the system and the real world’ and ‘Aesthetic and minimalist design’, issues concerned difficulty reading non-physiological data on line graphs, such as when patient medication had been changed. The issue under ‘User control and freedom’ concerned the relatively small size of the graphs, that did not re-size automatically, and which caused occasional difficulty in data interpretation. Positive comments were made about having detailed patient-level data displayed in general, and the use of tool-tips to understand historic physiological data on line graphs.

#### 5.1.4 Recommended actions

Issues associated with the recommended actions were categorised under the heuristics ‘Consistency and standards’ (n = 7), ‘Aesthetic and minimalist design’ (n = 4), ‘Error prevention and recognition’ (n = 3), ‘User control and freedom’ (n = 3), ‘Visibility of system status’ (n = 2), ‘Help and documentation’ (n = 1), and ‘Recognition rather than recall’ (n = 1). Under ‘Consistency and standards’, issues concerned conflicting use of language and font sizes, redundant column headers for user-generated actions, illogical ordering of options in dialogue boxes, and the positioning of recommended actions. Under ‘Aesthetic and minimalist design’, issues were deemed cosmetic issues only. Under ‘Error prevention and recognition’, issues concerned being able to edit a user-generated recommended action plan that had been marked “complete”, and technical faults related to deleting and downloading recommended actions (all rated as at least ‘major’ usability issues by 7 out of 8 evaluators). Under ‘User control and freedom’, issues concerned the inability to view, undo or edit reasons for disagreeing with recommended actions, or add user-generated recommended actions to the Home page. Under ‘Visibility of system status’ issues were: a lack of clarity as to whether marking a recommended action as complete had been saved by the system, and a loss of context when the dialogue box for providing disagreement reasons appeared. The issue under ‘Help and documentation’ recommended there should be some explanation of how the suggested actions were generated, whilst under ‘Recognition rather than recall’ it related to clearer signposting of the copy functionality for inputting patients’ unique identification numbers in other systems (e.g. EHRs). Positive comments were made about having the recommended actions in general, in addition to specific features including the interactive improvement opportunity graph to filter patient lists, ability to add user-generated actions, agree or disagree with actions, and provide reasons for disagreement in the form of both fixed responses (radio buttons) or more detailed free text.

### 5.2 Task and goal-Action structure

The 47 usability issues occurred 121 times in total across all tasks (median occurrences per issue of 2, range 1–12). In terms of both frequency and severity of usability issues, Task 4 (Disagree with patient identification) had the most usability violations (26 usability issue occurrences, 21% of the total). This was followed by Task 3 (Agree with patient-level action plan, n = 18, 15%), Task 2 (Disagree with team/organisation-level action plan, n = 16, 13%), Task 7 (Adding action plan, n = 15, 12%), Task 6 (Patient-level data identification, n = 14, 12%), and Task 5 (Population-level data identification, n = 12, 10%). Task 1 (Agree with team/organisation-level action plan) and Task 8 (General functionality) were the most issue-free with only 10 (8%) issues each. At the Goal-Action structure level (Appendix A in Supplementary material), the most issue-prone actions across all Goals were: (1) Patient selection from a list (which affected Goals 3, 4, and 6); (2) Data interpretation from a figure (both population-level and patient-level; Goals 5 and 6); and (3) Disagreement with a system proposition (this included: recommended actions [Goal 2] or categorisation of patients into an improvement opportunity group [Goal 4]). The remainder of this section describes how these actions impacted the completion of a given Goal or sub-Goal.

#### 5.2.1 Patient selection from a list

In Goals 3, 4, and 6, users navigated to the Overview page of the relevant clinical module (Fig. 1), and selected the summary of clinical performance to investigate further in the Preview page (Fig. 2a). Evaluators were then required to select a patient from the patient list either directly (Goals 4 and 6), or by first filtering the list using the improvement opportunity graph (Goal 3). At this point, the Goal-Action sequence was likely to be interrupted or become unwieldy due to: a lack of clarity as to what the different lists referred, and why they contained different patients; difficulty in browsing the patient lists due to a lack of visible ordering options and perceived lack of utility of the options by which they could be ordered; and an absence of feedback that a patient list had been filtered, or that different patient-level data was presented. To illustrate, in Goal 3 (Appendix A in Supplementary material) users would expect after selecting an improvement opportunity from the graph on the Preview page (action: ‘select palliative care’) that the filtering of the patient list would be apparent before proceeding to the following action (‘select patient 5556051664 from the list’). This issue was categorised under the heuristic ‘Visibility of System Status’, therefore making the status of the list in this part of the action sequence clearer would make the relationship between the two actions more natural for the user. The remaining issues related to the browsing and ordering of the list would make the process of completing the specific action of selecting a patient from a list less efficient, though were unlikely to disrupt the user’s action sequence.

#### 5.2.2 Data interpretation

In Goals 5 and 6, evaluators initially navigated either to the Overview (Fig. 1; to interpret population-level data) or Preview (Fig. 2a and b; to interpret patient-level data) pages respectively. In the Goal-Action structure, evaluators were then required to identify specific data points using the corresponding data visualisations (i.e. line graphs, bar plots, or pie-charts). At this point, the Goal-Action sequence was likely to be interrupted or hinder information processing due to: a lack of clarity regarding the specific aspects of clinical performance the graphs represented; the relatively small text size used for axis labels; unclear explanations for bar plot categories; and the use of non-standard date format (i.e. yyyy/mm/dd). Furthermore, interpreting patient-level non-physiological data (e.g. when medication had changed) were difficult because of misalignment with x-axis dates, an absence of tool-tips, and unclear labelling. To illustrate, in Goal 5, sub-Goal ‘identify how many patients have had face-to-face opportunities to have their asthma monitored’ (Appendix A in Supplementary material), users would expect to easily recognise precisely what the summary of clinical performance referred to on the Overview page (action: ‘select monitoring’) before proceeding to the following action (‘check the corresponding figure to identify how many patients have had face-to-face opportunities to have their asthma monitored’). This issue was categorised under the heuristic ‘Consistency and standards’, therefore making the specific aspects of clinical performance the graphs represented clearer would reduce the cognitive demands necessary for a user to understand how to access the relevant Preview page. The remaining issues made the identification of specific graph data points less efficient, though were unlikely to disrupt the prescribed action sequence.

#### 5.2.3 Disagreement with a system proposition

In Goals 2 and 4, users initially navigated to the Preview page of the relevant summary of clinical performance (Fig. 2a). At this point they were required to either disagree with an organisational-level action plan (Goal 2; Fig. 2a), or select a patient and disagree with the improvement opportunity to which it had been assigned (Goal 4; Fig. 2b). Despite the fact that several usability issues were identified in the context of recommended actions (Fig. 6), all of which could hinder the processing of information and increase the time needed to complete an action, none were likely to disrupt the Goal-Action sequence. To illustrate, in Goal 2 (Appendix A in Supplementary material) users would expect the recommended action plans to be in a conspicuous location and written in prominent font to facilitate their identification (action: ‘check the available option disagree for the action: “nominate an asthma lead … of these changes”’). Furthermore, they would expect to know how the recommended actions were generated in order to judge whether or not they agree. These issues were categorised under the heuristics ‘Consistency and standards’ and ‘Help and documentation’ respectively, therefore improved presentation of recommended actions, and provision of information regarding how they were generated would reduce the attentional and cognitive demands necessary to complete these Goals.

## 6 Discussion

Our results indicate important considerations that are specific e-A&F systems, and which should be taken into account in designing their interfaces. This final section discusses the significance of the usability issues found with PINGR, and translates them into a set of interface design recommendations for e-A&F systems in general by placing them in the context of the wider literature (Box 1). Each of the four components of e-A&F interfaces (summary of clinical performance, patient lists, patient-level data, and recommended actions) are considered in turn, followed by a final section on how they could be integrated. The paper concludes with a discussion of the strengths and weaknesses of this study, and implications for future research.

### 6.1 Interface design recommendations for e-A&F systems

#### 6.1.1 Summary of clinical performance

e-A&F system design should draw on existing usability guidance and theory for the presentation of clinical performance summaries [16], [31], in addition to relevant guidance on quantitative information visualisation in general (e.g. [38], [44]), and related IT systems including epidemiological surveillance tools (e.g. [12]) and non-clinical dashboards (e.g. [30]). Key recommendations include using line graphs to demonstrate trends over time [16], [31], [47], and interactive functionality to provide further detail on-demand [16], [44]. In addition, our results show that the use of tool-tips can facilitate accurate interpretation of historic performance data on line graphs, and that care should be taken to ensure what performance data specifically refers. If this is not the case, users may disengage with the system, with potentially important implications for patient outcomes and resource-use (e.g. [35]).

#### 6.1.2 Patient lists

Not all e-A&F systems provide lists of patients (e.g. [5]), despite evidence from non-computerised A&F interventions suggesting they are key drivers of success [26], [27]. Therefore, a key recommendation is to include patient lists as a core part of e-A&F interface design. These may include patients who have or have not achieved the quality standard or patient outcome of interest. The design of patient lists may utilise existing evidence from non-computerised A&F interventions [26], [27], in addition to usability guidelines from related health systems such as EHRs (e.g. [39], [49]) and epidemiological surveillance tools (e.g. [29]). Key recommendations include the ability to order and prioritise patients for action [27], [39], and providing a manageable number of variables by which to order patients [29]. Our results add that the use of visual querying mechanisms to filter lists, and the ability to order lists can also be helpful, which is supported by studies of other e-A&F systems and wider usability guidelines [16], [44]. However, our results also highlight that it should be apparent that lists have changed when they are filtered, thus providing clear feedback of system status. This may be achieved through the use of animation (e.g. self-healing fades) and significant changes to the text in the list header. Furthermore, enough information should be provided regarding what the lists refer to, in addition to making the ordering functionality obvious and using parameters perceived as valuable by users. If patient lists are not designed in a usable way, it may force users to identify patients in an inefficient manner, leading to reduced system effectiveness or disruption of the Goal-Action structure.

#### 6.1.3 Patient-level data

Not all e-A&F systems provide patient-level data (e.g. [23]), therefore a key recommendation from our results is to include this interface component as a core design consideration. This is supported by other studies of e-A&F systems [16], and ensures the system can efficiently support improvement action [25], [27]. The design of this component should draw on existing usability knowledge [16], in addition to design guidelines for systems that summarise individual patient-level data, such as EHRs (e.g. [50]). Important recommendations include the use of line graphs to support trend visualisation over defined time periods, and the ability to interactively explore data further [16]. Our results also suggest that similar to population-level data, tool-tips can be helpful to understand historic data. In addition, provision of non-physiological data, such as contacts with the primary care practice or changes in medication, can improve feedback actionability, though must be displayed as clearly as the medical data to be effective. This could be achieved through novel information visualisation techniques such as Lifelines [79]. Finally, our results also illustrate the importance of highlighting that new patient-level data is displayed when a new patient is selected (e.g. from a list). If this is unclear users may be unsure how to access relevant patient-level data and therefore unable to take relevant improvement action. This may be remedied through the use of animation, or other design features such as presenting individual patient’s data on separate pages from each other.

#### 6.1.4 Recommended actions

In addition to PINGR, we are aware of only one other e-A&F system that provides recommended actions to users [23]. This is surprising given such recommendations are part of the definition of A&F [2], that there is both theoretical [31] and empirical evidence [1] they increase A&F effectiveness, and that user-needs assessments for e-A&F systems state they are desirable [16], [23]. Our results confirm this need, therefore a key design recommendation is that e-A&F systems should provide recommended actions; creating a cross-fertilisation of traditional A&F and CDS systems [41]. The design of recommended actions should draw on guidance regarding CDS systems that regularly provide advice to users (e.g. [40]) and wider quality improvement theory (e.g. [51]). Specifically, recommended actions should address both the individual patient and organisation [51], be specific to user’s context and performance (rather than generic) [40], be provided automatically in the user’s workflow (rather than on-demand) [40], take into account patient contextual data (such as co-morbidities) [53], [56], and use concise statements [40], [53] with functionality to easily action the recommendation [40], [53]. Furthermore, our findings suggest that the ability to agree (and save) or disagree (and provide fixed and free-text response reasons) with recommended actions is well-received by users, which is supported by the wider CDS system literature [53], [56], and may improve e-A&F effectiveness [57]. This feedback loop should be used to improve the algorithms driving the e-A&F system [25], [80]. Our results also suggest that functionality to action recommendations is made clear, such as using tool-tips to highlight a ‘copy’ function, otherwise their effectiveness may be reduced. Information should also be provided on how the recommendation was generated [40], [56], and users provided with unambiguous and authoritative confirmation that their agreements or disagreements with the recommendation have been saved, otherwise there is risk users would find the system untrustworthy, and potentially ignore the recommendations. Mechanisms for providing disagreement reasons should not lose context from the recommended action, and there should be functionality to view, undo and edit reasons, otherwise users may disengage with this functionality and the gains in algorithm improvement would be lost. However, editing should not be possible once an action is marked complete, otherwise users may fear their work is lost. Recommended actions should be positioned in their own area separate from other data, using the same size font as other information, and in an area of the interface consistent with user workflow (which may not be the bottom of the page). This mitigates the risk they will be overlooked. Finally, users should also have the ability to add their own recommended actions, which should be available throughout the system where actions are displayed.

#### 6.1.5 Integration of interface components

Interface components should be integrated in a way consistent with general software design guidelines (e.g. [46]), in addition to related health IT systems such as CDS systems (e.g. [53], [56]) and EHRs (e.g. [39]). For example, components should be arranged in a way that anticipates user’s workflow [48], and patient-level data should be presented on the same screen as recommended actions so they can reliably be evaluated [53], [56]. EHR usability guidelines recommend that patient-level information should not be on the same page as patient lists [49], however, we suggest this may not be applicable to e-A&F systems because of the need to rapidly review multiple patients, and an absence of e-A&F functionality related to data input (unlike an EHR). It is also unclear whether patient-level data should be displayed on the same screen as summaries of clinical performance (as in PINGR), as there is a theoretical argument it may motivate action regarding a single patient if a user is made aware of their wider performance [41].

### 6.2 Strengths and limitations of this study

The main strength of this study is that it is the first to develop usability recommendations for clinical e-A&F systems. This was achieved by evaluating a cutting-edge e-A&F system, which to the best of our knowledge is the first to contain all four key e-A&F interface elements (summaries of clinical performance, patient lists, patient-level data, and recommended actions), and whose design was informed by existing relevant usability principles and theory. We used an innovative approach to usability inspection that combined the strengths of both HE and CW. This meant we could evaluate not only the individual interface components (as in an HE; Objective 2), but also how they should be integrated into a single system to support the Goal-Action structure of typical tasks (Objective 3). Combining these two methods maximised usability issue discovery, which was further helped by using eight evaluators working independently rather than the widely recommended three to five in traditional usability inspections [64], [65]. The development and use of a thorough usability inspection protocol was important given the relative lack of knowledge in the wider literature regarding usability of e-A&F systems.

The main limitation of this study is that results are based on a usability inspection with expert evaluators, rather than testing with representative end-users (primary care clinicians) [34]. This may result in a number of the issues identified being false positives [70], or potentially missing important issues that may only be apparent with further tests in more naturalistic settings [34]. For example, end-users may not consider being able to edit an action plan marked “complete” a usability issue, as they may wish to retrospectively clarify events; conversely, although end-users may consider the display of actions recommended by PINGR *usable*, they may not find them *useful* in improving patient care. Furthermore, expert evaluators may be able to navigate the system more efficiently than target end-users. Specific actions were taken to minimise the impact of these limitations, such as the use of a background document describing the characteristics of intended users (Fig. 4), and a thorough hybrid inspection method with multiple data collection and analysis stages, which included a usability issue consolidation stage to ensure the most important unique usability issues were analysed. Nevertheless, our findings should be interpreted with caution, and we consequently deem our usability recommendations for e-A&F systems as ‘preliminary’. To further address these shortcomings, and in accordance with accepted usability engineering methodology [34], we plan to undertake further evaluations of PINGR with target end-users in future.

Further limitations are that our hybrid usability inspection method mainly focused on identification of heuristic violations for each action, rather than the detailed reconstruction of a user’s cognitive goal as in traditional CW [66]. For example, a traditional CW may ask the evaluator to estimate the percentage of users that will perform a specific action, the level of agreement between a given and actual user’s goal, or the likelihood that users’ goals will change after the performance of an action [66]. However, incorporating all these elements would likely make the hybrid method too cumbersome, which is a criticism of the traditional CW method [81].

Finally, we assessed inter-rater variation, which is recommended for usability inspection studies [82], and can therefore be viewed as a methodological strength. We found weak-moderate agreement and poor-fair reliability between evaluator’s issue severity ratings, which may reflect a useful variety of raters’ abilities to detect a wide variety of potential issues, though conversely may also be viewed as a limitation of our results. However, low levels of agreement are common in usability inspection studies [67], and it becomes increasingly hard to achieve agreement as the number of raters increases [71]. Usually three to five evaluators are recommended for usability inspection studies [64], [65], though we used eight in order to detect as many unique issues as possible. To demonstrate, our IRA and IRR metrics improve if re-calculated using the ratings of only three instead of eight evaluators: complete agreement = 32%, Kendall’s coefficient = 0.68, ICC = 0.47, and Weighted Light’s Kappa = 0.46; Krippendorff's alpha remains at 0.04, which is consistent with a comparable study [82]. Therefore, our relatively low levels of agreement and reliability are a necessary consequence of attempting to achieve our study objectives.

## 7 Conclusion

We have presented the usability evaluation of a modern, research-led clinical e-A&F system (PINGR) using a hybrid usability inspection method. In doing so, we described its design, rationale and theoretical basis (Objective 1), identified usability issues in relation to its four interface components (summaries of clinical performance, patient lists, patient-level data, and recommended actions; Objective 2), and attempted to understand how these issues may interfere with the cognitive goals of end-users of the system (Objective 3). Based on our findings and the wider literature, we have developed a set of recommendations for the user-centred design of e-A&F systems that addresses key interface components, in addition to how they should be integrated (Study objective 4). These recommendations go some way to addressing the gaps in the literature regarding the optimal design of clinical e-A&F systems. Future research should refine and extend this much needed evidence base.

**Summary points**

What was already known on the topic

• Audit and Feedback (A&F) is a widely used quality improvement technique that measures clinicians’ performance and reports it to them.
• There is a lack of evidence regarding how to best design computerised A&F (e-A&F) system interfaces.

What this study added to our knowledge

• Establishes that e-A&F system interfaces may consist of four key components: (1) Summaries of clinical performance; (2) Patient lists; (3) Patient-level data; (4) Recommended actions.
• Demonstrates how the design of e-A&F systems can be based on existing evidence and theory.
• Introduces an innovative hybrid inspection approach that combines the advantages of both Heuristic Evaluation and Cognitive Walkthrough methods.
• Presents recommendations for the user-centred design of e-A&F systems in terms of their four key interface components, and how they may be integrated within a system.

## Author contributions

All authors contributed to the conception and design of the study. BB conceived and designed PINGR, which was built by both RW and BB. BB and PB collected and analysed the data. All authors contributed to and approved the final version of the manuscript.

## Funding

This work was supported by a Wellcome Trust Research Training Fellowship for BB [104438/Z/14/Z], and the MRC Health e-Research Centre, Farr Institute of Health Informatics Research [MR/K006665/1].

## Competing interests

Nil.

## Author contributions

All authors contributed to the conception and design of the study. BB conceived and designed PINGR, which was built by both RW and BB. BB and PB collected and analysed the data. All authors contributed to and approved the final version of the manuscript.

## Figures and Tables

**Table 1:** Overview of tasks performed by evaluators during usability inspection.

Number	Brief description of task and Goal	Interface components assessed
1	Agree with team/organisation-level action plan	Menu Clinical performance summary Recommended actions
2	Disagree with team/organisation-level action plan	Menu Clinical performance summary Recommended actions
3	Agree with patient-level action plan	Menu Clinical performance summary Patient lists Recommended actions
4	Disagree with patient identification	Menu Clinical performance summary Patient lists Patient-level data
5	Population-level data intepretation	Menu Clinical performance summary
6	Patient-level data intepretation	Menu Clinical performance summary Patient lists Patient-level data
7	Adding action plan	Menu Clinical performance summary Recommended actions
8	General functionality^a^	Search box Patient lists Recommended actions

a Included: searching for a specific patient, ordering the lists of patients according to specific criteria, and downloading a summary of activity/actions made using the PINGR system.

**Table 2:** Usability issue severity rating scale.

Rating	Description
1	Cosmetic issue only. Need not be fixed unless extra time is available on project
2	Minor usability issue. Fixing this should be given low priority
3	Major usability issue. Important to fix, so should be given high priority
4	Usability catastrophe. Imperative to fix this before product can be released

## Box 1 Interface design recommendations and future research questions for computerised audit and feedback systems.

Summaries of clinical performance, should:

• Use line graphs to demonstrate trends over time
• Provide interactive functionality and further detail on-demand
• Use tool-tips to interpret historic performance data
• Explain clearly to what performance data specifically refer

Patient lists, should:

• Be included as a key component of the e-A&F interface
• Provide ordering functionality that is clearly labelled
• Use a manageable number of variables (considered valuable by users) by which to order patients
• Use visual querying mechanisms to filter contents, providing clear indication when the list changes
• Explain clearly to what the lists refer

Patient-level data, should:

• Be included as a key component of the e-A&F interface
• Use line graphs to monitor trends over time
• Provide interactive functionality to explore
• Use tool-tips to interpret historic datadata
• Include relevant non-clinical data
• Indicate clearly when a new patient’s data is displayed

Recommended actions, should:

• Address both the individual patient and organisation
• Be specific to a user’s context and performance (rather than generic)
• Be provided automatically in the user’s workflow (rather than on-demand)
• Take into account patient contextual data (such as co-morbidities)
• Use concise statements
• Provide functionality to clearly and easily action the recommendation
• Provide functionality to agree (and save) or disagree with recommendations
• Collect reasons for disagreement as both fixed and free-text responses
• Use disagreement reasons to improve system algorithms
• Provide information on how recommendations were generated
• Provide unambiguous and authoritative confirmation that agreements or disagreements with recommendations have been saved
• Maintain context when asking for disagreement reasons
• Provide functionality to view, undo and edit previous reasons given for disagreements
• Not allow editing once an action is marked complete
• Be positioned in a separate area of the interface, consistent with the anticipated user workflow
• Use the same size font as other information
• Provide functionality to add user-generated recommended actions throughout the system

Integration of components:

• Components should be arranged in a way that anticipates user’s workflow
• Patient-level data should be presented on the same screen as recommended actions

Questions for further research:

• What interface components should be visible at the same time?
• Which patients should be listed and how much information about them should be provided in the lists?
• How should clinical performance summaries and recommended actions be prioritised?
• How should patient-level data across multiple clinical areas and quality indicators be presented?
• How should the clinical performance of other users be incorporated, if at all?
• How do these guidelines translate into different contexts, clinical areas, and study designs?
• Do these guidelines remain true when testing with real end-users?

Alt-text: Box 1

## References

[1] Ivers N., Jamtvedt G., Flottorp S., Jm Y., Sd F., Ma O.B. (2012). Audit and feedback: effects on professional practice and healthcare outcomes. Cochrane Database Syst. Rev..

[2] EPOC, Data Collection Checklist, Cochrane Eff. Pract. Organ. Care Rev. Gr. 2002.

[3] Ross Scrivener C., Morrell C., Baker R., Redsell S., Shaw E., Stevenson K. (2002). Principles for Best Practice in Clinical Audit.

[4] HQIP, Criteria and indicators of best practice in clinical audit, London, 2009.

[5] Geller B.M., Ichikawa L., Miglioretti D.L., Eastman D. (2012). Web-based mammography audit feedback. Am. J. Roentgenol..

[6] Linder J.A., Schnipper J.L., Tsurikova R., Yu D.T., Volk L.A., Melnikas A.J. (2010). Electronic health record feedback to improve antibiotic prescribing for acute respiratory infections. Am. J. Manage. Care.

[7] Colquhoun H., Michie S., Sales A., Ivers N., Grimshaw J.M., Carroll K. (2016). Reporting and design elements of audit and feedback interventions: a secondary review. BMJ Qual. Saf..

[8] Avery A.J., Rodgers S., Cantrill J.A., Armstrong S., Cresswell K., Eden M. (2012). A pharmacist-led information technology intervention for medication errors (PINCER): a multicentre, cluster randomised, controlled trial and cost-effectiveness analysis. Lancet.

[9] Dowding D., Randell R., Gardner P., Fitzpatrick G., Dykes P., Favela J. (2015). Dashboards for improving patient care: review of the literature. Int. J. Med. Inform..

[10] Koopman R.J., Kochendorfer K.M., Moore J.L., Mehr D.R., Wakefield D.S., Yadamsuren B. (2011). A diabetes dashboard and physician efficiency and accuracy in accessing data needed for high-quality diabetes care. Ann. Fam. Med..

[11] Zaydfudim V., Dossett L.A., Starmer J.M., Arbogast P.G., Feurer I.D., Ray W.A. (2009). Implementation of a real-time compliance dashboard to help reduce SICU ventilator-associated pneumonia with the ventilator bundle. Arch. Surg..

[12] Robinson A.C., Roth R.E., MacEachren A.M. (2011). Designing a web-Based learning portal for geographic visualization and analysis in public health. Heal. Inf. J..

[13] Curtis J.R., Westfall A.O., Allison J., Becker A., Melton M.E., Freeman A. (2007). Challenges in improving the quality of osteoporosis care for long-term glucocorticoid users: a prospective randomized trial. Arch. Intern. Med..

[14] Itri J.N., Jones L.P., Kim W., Boonn W.W., Kolansky A.S., Hilton S. (2014). Developing an automated database for monitoring ultrasound- and computed tomography-guided procedure complications and diagnostic yield. J. Digit Imaging.

[15] Ratwani R.M., Fong A. (2015). Connecting the dots: leveraging visual analytics to make sense of patient safety event reports. J. Am. Med. Inform. Assoc..

[16] A.L. Hartzler, B.C., Fey, D.R. Flum, Integrating Patient-Reported Outcomes into Spine Surgical Care through Visual Dashboards: Lessons Learned from Human-Centered Design Integrating Patient-Reported Outcomes into Spine Surgical Care through, 3 (2015) 3–13. doi: 10.13063/2327-9214.1133. 10.13063/2327-9214.1133 PMC4431498 25988187

[17] Brown B., Jameson D., Daker-White G., Buchan I., Ivers N., Peek N. (2015). A meta-synthesis of findings from qualitative studies of audit and feedback interventions. PROSPERO Int. Prospect. Regist. Syst. Rev..

[18] Mainz J. (2003). Defining and classifying clinical indicators for quality improvement. Int. J. Qual. Health Care.

[19] Lo Y.-S., Lee W.-S., Chen G.-B., Liu C.-T. (2014). Improving the work efficiency of healthcare-associated infection surveillance using electronic medical records. Comput. Methods Programs Biomed..

[20] Guldberg T.L., Vedsted P., Kristensen J.K., Lauritzen T. (2011). Improved quality of Type 2 diabetes care following electronic feedback of treatment status to general practitioners: a cluster randomized controlled trial. Diabet. Med..

[21] Daley K., Richardson J., James I., Chambers A., Corbett D. (2013). Clinical dashboard: use in older adult mental health wards. Psychiatrist.

[22] Morgan M.B., Branstetter B.F., Lionetti D.M., Richardson J.S., Chang P.J. (2008). The radiology digital dashboard: effects on report turnaround time. J. Digit Imaging.

[23] Meijers J.M.M., Halfens R.J.G., Mijnarends D.M., Mostert H., Schols J.M.G.A. (2013). A feedback system to improve the quality of nutritional care. Nutrition.

[24] Waitman L.R., Phillips I.E., McCoy A.B., Danciu I., Halpenny R.M., Nelsen C.L. (2011). Adopting real-time surveillance dashboards as a component of an enterprisewide medication safety strategy. Jt. Comm. J. Qual. Patient Saf..

[25] Grant A.M., Guthrie B., Dreischulte T. (2014). Developing a complex intervention to improve prescribing safety in primary care: mixed methods feasibility and optimisation pilot study. BMJ Open.

[26] Cresswell K.M., Sadler S., Rodgers S., Avery A., Cantrill J., Murray S.A. (2012). An embedded longitudinal multi-faceted qualitative evaluation of a complex cluster randomized controlled trial aiming to reduce clinically important errors in medicines management in general practice. Trials.

[27] Ivers N., Barnsley J., Upshur R., Tu K., Shah B., Grimshaw J. (2014). My approach to this job is…one person at a time. Can. Fam. Physician.

[28] Pagliari C., Clark D., Hunter K., Boyle D., Cunningham S., Morris A. (2003). DARTS 2000 online diabetes management system: formative evaluation in clinical practice. J. Eval. Clin. Pract..

[29] De Croon R., Klerkx J., Duval E. (2015). Design and evaluation of an interactive proof-of-concept dashboard for general practitioners. 2014 IEEE Int. Conf. Healthc. Informatics.

[30] Yigitbasioglu O.M., Velcu O. (2012). A review of dashboards in performance management: implications for design and research. Int. J. Account. Inf. Syst..

[31] Kluger A.N., DeNisi A. (1996). The effects of feedback interventions on performance: a historical review, a meta-analysis, and a preliminary feedback intervention theory. Psychol. Bull..

[32] Tjon W., Sjoe S., van Der Zwan E.P.A., Peek N. (2013). A Web-based System to Facilitate Local, Systematic Quality Improvement by Multidisciplinary Care Teams: Development and First Experiences of CARDSS Online.

[33] Marcilly R., Ammenwerth E., Roehrer E., Pelayo S., Vasseur F. (2015). Usability flaws in medication alerting systems: impact on usage and work system. Yearb. Med. Inform..

[34] Borycki E., Kushniruk A., Nohr C., Takeda H., Kuwata S., Carvalho C. (2013). Usability methods for ensuring health information technology safety: evidence-Based approaches. contribution of the IMIA working group health informatics for patient safety. Yearb. Med. Inform..

[35] Francis R. (2013). Report of the Mid Staffordshire NHS Foundation Trust Public Inquiry Executive Summary Report of the Mid Staffordshire NHS Foundation Trust Public Inquiry.

[36] Simpao A.F., Ahumada L.M., Desai B.R., Bonafide C.P., Galvez J.A., Rehman M.A. (2014). Optimization of drug–drug interaction alert rules in a pediatric hospital’s electronic health record system using a visual analytics dashboard. J. Am. Med. Inf. Assoc..

[37] Vaishnavi V.K., Kuechler W. (2015). Design Science Research Methods and Patterns: Innovating Information and Communication Technology.

[38] Tufte E.R. (2001). The Visual Display of Quantitative Information.

[39] Zahabi M., Kaber D.B., Swangnetr M. (2015). Usability and safety in electronic medical records interface design: a review of recent literature and guideline formulation. Hum. Factors J. Hum. Factors Ergon. Soc..

[40] Horsky J., Schiff G.D., Johnston D., Mercincavage L., Bell D., Middleton B. (2012). Interface design principles for usable decision support: a targeted review of best practices for clinical prescribing interventions. J. Biomed. Inform..

[41] Brown B., Peek N., Buchan I. (2015). The case for conceptual and computable cross-Fertilization between audit and feedback and clinical decision support. Stud Health Technol. Inform..

[42] Scott-Wright A., Fischer R., Denekamp Y., Boxwala A. (2004). A methodology for modular representation of guidelines. Stud. Health Technol. Inf..

[43] Schwabe D., Rossi G. (1998). An object oriented approach to web-based applications design. Theor. Pract. Object Syst..

[44] Shneiderman B. (1996). The eyes have it: a task by data type taxonomy for information visualizations. Proc. 1996 IEEE Symp. Vis. Lang..

[45] Edwards A., Elwyn G., Covey J., Matthews E., Pill R. (2001). Presenting risk information − a review of the effects of framing” and other manipulations on patient outcomes. J. Health Commun..

[46] Shneiderman B., Plaisant C. (2004). Designing the User Interface: Strategies for Effective Human-computer Interaction.

[47] Vessey I. (1991). Cognitive fit: a theory-based analysis of the graph versus tables literature. Decis. Sci..

[48] Nielsen J. (2006). F-Shaped Pattern For Reading Web Content.

[49] Microsoft, Patient List View, in: Des Guid., 2015.

[50] Displaying Microsoft Graphs and Tables, in: Des Guid., 2008.

[51] Ferlie E., Shortel S. (2000). Improving the quality of health care in the United Kingdom and the United States: a framework for change. Milbank Q..

[52] Bates D.W., Kuperman G.J., Wang S., Gandhi T., Kittler A., Volk L. (2003). Ten commandments for effective clinical decision support: making the practice of evidence-based medicine a reality. J. Am. Med. Inform. Assoc..

[53] Horsky J., Phansalkar S., Desai A., Bell D., Middleton B. (2013). Design of decision support interventions for medication prescribing. Int. J. Med. Inform..

[54] Glynn L.G., Murphy A.W., Smith S.M., Schroeder K., Fahey T. (2010). Interventions used to improve control of blood pressure in patients with hypertension. Cochrane Database Syst. Rev..

[55] Brown B., Williams R., Sperrin M., Frank T., Ainsworth J., Buchan I. (2014). Making audit actionable: an example algorithm for blood pressure management in chronic kidney disease. AMIA Annu. Symp. Proc..

[56] Marcilly R., Ammenwerth E., Vasseur F., Roehrer E., Beuscart-Zephir M.C. (2015). Usability flaws of medication-related alerting functions: a systematic qualitative review. J. Biomed. Inf..

[57] Roshanov P.S., Fernandes N., Wilczynski J.M., Hemens B.J., You J.J., Handler S.M. (2013). Features of effective computerised clinical decision support systems: meta-regression of 162 randomised trials. BMJ.

[58] Nielsen J. (1994). Usablity Inspection Methods.

[59] Kamper R.J. (2002). Extending the usability of heuristics for design and evaluation: lead, follow, and get out of the way. Int. J. Hum. Comput. Interact..

[60] Carvalho C.J., Borycki E.M., Kushniruk A. (2009). Ensuring the safety of health information systems: using heuristics for patient safety. Healthcare Q..

[61] Kushniruk A.W., Monkman H., Tuden D., Courtney K.L., Kuo A., Shabestari O. (2015). Integrating heuristic evaluation with cognitive walkthrough: development of a hybrid usability inspection method. Driv. Qual. Informatics Fulfilling Promise K.L..

[62] Sears A. (1997). Heuristic walkthroughs: finding the problems without the noise. Int. J. Hum. Comput. Interact..

[63] Andre T.S., Hartson H.R., Williges R.C. (2003). Determining the effectiveness of the usability problem inspector: a theory-based model and tool for finding usability problems. Hum. Factors.

[64] Nielsen J. (1992). Finding usability problems through heuristic evaluation. Proc. SIGCHI Conf. Hum..

[65] Jacobsen N.E., Hertzum M., John B.E. (1998). The evaluator effect in usability studies: problem detection and severity judgments. Proc. Hum. Factors Ergon. Soc. Annu. Meet..

[66] Polson P.G., Lewis C., Rieman J., Wharton C. (1992). Cognitive walkthroughs: a method for theory-based evaluation of user interfaces. Int. J. Man. Mach. Stud..

[67] Hertzum M., Jacobsen N.E. (2003). The evaluator effect: a chilling fact about usability evaluation methods. Int. J. Hum. Comput. Interact..

[68] Nielsen J. (1993).

[69] Nielsen J. (1995). 10 Heuristics for User Interface Design. http://www.nngroup.com/articles/ten-usability-heuristics/.

[70] Jeffries R., Miller J.R., Wharton C., Uyeda K. (1991). User interface evaluation in the real world: a comparison of four techniques. Proc. SIGCHI Conf. Hum. Factors Comput. Syst. Reach. through Technol. − CHI ’91.

[71] Gisev N., Bell J.S., Chen T.F. (2013). Interrater agreement and interrater reliability: key concepts, approaches, and applications. Res. Soc. Adm. Pharm..

[72] R Core Team R. A language and environment for statistical computing. Vienna, 2014.

[73] M. Gamer, Package “irr” v0.84: Various Coefficients of Interrater Reliability and Agreement, Hamburg, 2015.

[74] B. Falissard, Package psy v1.1: Various procedures used in psychometry, 2015.

[75] Gisev N., Bell J.S., O’Reilly C.L., Rosen A., Chen T.F. (2010). An expert panel assessment of comprehensive medication reviews for clients of community mental health teams. Soc. Psychiatry Psychiatr. Epidemiol..

[76] Schmidt R.C. (1997). Managing delphi surveys using nonparametric statistical techniques*. Decis. Sci..

[77] K. Krippendorff, Content Analysis: An introduction to its methodology, Thousand Oaks, London, 1980.

[78] R. Hernaez, Reliability and agreement studies: a guide for clinical investigators 2015. 10.1136/gutjnl-2014-308619 25873640

[79] Rind A., Wang T.D., Aigner W., Miksch S., Wongsuphasawat K., Plaisant C. (2011). Interactive information visualization to explore and query electronic health records. Found. Trends Human-Computer Interact..

[80] Payne T.H., Hines L.E., Chan R.C., Hartman S., Kapusnik-Uner J., Russ A.L. (2015). Recommendations to improve the usability of drug-drug interaction clinical decision support alerts. J. Am. Med. Inform. Assoc..

[81] Spencer R. (2000). The streamlined cognitive walkthrough method, working around social constraints encountered in a software development company. Proc. SIGCHI Conf. Hum. Factors Comput. Syst..

[82] Georgsson M., Weir C.R., Lovis C., Séroussi B., Hasman A., Pape-Haugaard L., Saka O., Andersen S.K. (2014). Revisiting heuristic evaluation methods to improve the reliability of findings. E-Health Contin. Care.

