# Supplementary material for: Interface design recommendations for computerised clinical audit and feedback: Hybrid usability evidence from a research-led system
Source: Int J Med Inform. 2016 Oct;94:191–206. doi: 10.1016/j.ijmedinf.2016.07.010 (PMC5015594; doi:10.1016/j.ijmedinf.2016.07.010)
Supplement: Supplementary file 1 [file mmc1.docx]

**Appendix A: User tasks and Goal-Action structure provided to system evaluators**

**Task 1**

AGREE WITH THE SUGGESTED ACTION PLAN FOR MONITORING BLOOD PRESSURE: “INTRODUCE A TEXT-MESSAGING SERVICE TO REMIND PATIENTS THEY NEED A BLOOD PRESSURE CHECK”

SELECT BLOOD PRESSURE

and then SELECT MONITORING

and then CHECK THE AVAILABLE OPTION “AGREE” FOR THE ACTION: “INTRODUCE … BLOOD PRESSURE CHECK”

and then CHECK THE AVAILABLE OPTION TO MARK THIS ACTION AS COMPLETED

**Task 2**

DISAGREE WITH THE SUGGESTED ACTION PLAN FOR TREATING ASTHMA: “NOMINATE AN ASTHMA LEAD IN YOUR PRACTICE WHO CAN INITIATE SOME OF THESE CHANGES”

SELECT ASTHMA

and then SELECT TREATMENT

and then CHECK THE AVAILABLE OPTION DISAGREE FOR THE ACTION: “NOMINATE AN ASTHMA LEAD … OF THESE CHANGES”

and then TYPE IN THE AVAILABLE FORM THE RESPONSE “ALREADY DONE THIS”

**Task 3**

AGREE WITH THE SUGGESTED ACTION PLAN “ADD CODE ‘9H31’ (PATIENT UNSUITABLE)” FOR PATIENT 5556051664 WITH HIGH BLOOD PRESSURE, CURRENTLY RECEIVING PALLIATIVE CARE, WHO MAY BENEFIT FROM BEING EXCLUDED FROM QUALITY STANDARDS.

SELECT BLOOD PRESSURE

and then SELECT EXCLUSIONS

and then SELECT PALLIATIVE CARE

and then SELECT PATIENT 5556051664 FROM THE LIST

and then CHECK THE AVAILABLE OPTION AGREE FOR THE ACTION “ADD CODE ‘9H31’ (PATIENT IS UNSUITABLE)”

and then COPY THE CODE ‘9H31’

and then PASTE THE CODE ‘9H31’ INTO THE SEPARATE EHR APPLICATION

and then CHECK THE AVAILABLE OPTION TO MARK THIS ACTION AS COMPLETED

**Task 4**

ASSESS WHETHER THE APPLICATION HAS IDENTIFIED THE CORRECT IMPROVEMENT OPPORTUNITIES AND QUALITY STANDARDS FOR PATIENT 6662563783 WHO’S ASTHMA IS BEING MONITORED

SELECT ASTHMA

and then SELECT MONITORING

and then SELECT PATIENT 6662563783

and then COPY THE PATIENT’S NUMBER INTO THE SEPARATE ‘EHR’

DISAGREE WITH AND CORRECT THE IMPROVEMENT OPPORTUNITY “NON FACE-TO-FACE” THAT THE APPLICATION HAS CATEGORISED THE PATIENT 6662563783 WHO’S ASTHMA IS BEING MONITORED

CHECK THE AVAILABLE OPTION DISAGREE FOR THE IMPROVEMENT OPPORTUNITY ‘NON FACE-TO-FACE

and then TYPE IN THE AVAILABLE FORM THE CORRECT ACTION ‘SHOULD BE IN THE NO OPPORTUNITIES GROUP’

DISAGREE WITH AND CORRECT THE QUALITY STANDARD ‘ANNUAL REVIEW’ THAT THE APPLICATION SUGGESTS THAT THE SAME PATIENT HAS MISSED

CHECK THE AVAILABLE OPTION DISAGREE FOR THE QUALITY STANDARD ‘ANNUAL REVIEW MISSED’

and then TYPE IN THE AVAILABLE FORM THE CORRECT STATUS FOR THE PATIENT ‘HAD REVIEW AT WORK’

**Task 5**

IDENTIFY SOME DESCRIPTIVE STATISTICS AT THE POPULATION LEVEL ABOUT THE CARE PROVIDED IN YOUR PRACTICE FOR PATIENTS WITH ASTHMA

IDENTIFY HOW MANY PATIENTS HAVE BEEN EXCLUDED FROM AASTHMA QUALITY STANDARDS FOR ‘REASOSNS WE THINK’

SELECT ASTHMA

and then CHECK THE CORRESPONDING FIGURE TO IDENTIFY HOW MANY PATIENTS HAVE BEEN EXCLUDED FROM ASTHMA QUALITY STANDARDS FOR ‘REASONS WE THINK’

IDENTIFY THE PERCENTAGE OF PATIENTS THAT HAD MONITORED ASTHMA ON 1^ST^ APRIL 2015

CHECK THE CORRESPONDING FIGURE TO IDENTIFY THE PERCENTAGE OF PATIENTS THAT HAD MONITORED ASTHMA ON 1^ST^ APRIL 2015

IDENTIFY HOW MANY PATIENTS HAVE HAD FACE-TO-FACE OPPORTUNITIES TO HAVE THEIR ASTHMA MONITORED

SELECT MONITORING

and then CHECK THE CORRESPONDING FIGURE TO IDENTIFY HOW MANY PATIENTS HAVE HAD FACE-TO-FACE OPPORTUNITIES TO HAVE THEIR ASTHMA MONITORED

Task 6

IDENTIFY SPECIFIC INFORMATION ABOUT THE BLOOD PRESSURE TREATMENT PROVIDED TO PATIENT 5554632673. SPECIFICALLY YOU NEED TO FIND WHAT HIS/HER BLOOD PRESSURE READING WAS ON THE 12^TH^ SEPTEMBER 2013 AND WHAT DATE WAS HIS/HER BLOOD PRESSURE MEDICATION INCREASED.

IDENTIFY WHAT THE BLOOD PRESSURE READING OF PATIENT 5554632673 WAS ON 12^TH^ SEPTEMBER 2013

SELECT BLOOD PRESSURE

and then SELECT TREATMENT

and then SELECT PATIENT 5554632673

and then CHECK THE AVAILABLE FIGURE TO IDENTIFY HIS /HER BLOOD PRESSURE READING ON 12^TH^ SEPTEMBER 2013

IDENTIFY WHAT WAS THE DATE WHEN THE BLOOD PRESSURE MEDICATION OF PATIENT 5554632673 WAS INCREASED

CHECK THE AVAILABLE FIGURE TO IDENTIFY THE DATE HIS/HER BLOOD PRESSURE MEDICATION WAS INCREASED

**Task 7**

ADD AND EDIT YOUR OWN TEAM/ORGANISATION ACTION PLANS FOR BLOOD PRESSURE MONITORING

SELECT BLOOD PRESSURE

and then SELECT MONITORING

and then ENTER YOUR OWN ACTION PLAN: “EMPLOY AN ADDITIONAL NURSE” AT THE TEAM/ORGANISATION TAB

and then CHECK THE AVAILABLE OPTION TO DENOTE THAT THIS ACTION HAS BEEN COMPLETED

and then ENTER ANOTHER ACTION PLAN : “ADD ADDITIONAL SURGERIES”

and then EDIT THIS NEW ACTION PLAN TO SAY: “ADD SATURDAY SURGERIES”

and then DELETE THE ACTION PLAN : “ADD SATURDAY SURGERIES”.

TASK 8 (the purpose of this task was to test some general features of the functionality of the PINGR system. These were not tested as part of a goal – action structure because they represented only atomic actions that in our case did not take place in the context of a broader goal).

SEARCH FOR PATIENT 5557989507

ORDER THE LIST OF PATIENTS BY THEIR LATEST SBP

SWITCH QUALITY STANDARDS

DOWNLOAD YOUR ACTION PLAN FOR PRINTING
