# Supplementary material for: Interface design recommendations for computerised clinical audit and feedback: Hybrid usability evidence from a research-led system
Source: Int J Med Inform. 2016 Oct;94:191–206. doi: 10.1016/j.ijmedinf.2016.07.010 (PMC5015594; doi:10.1016/j.ijmedinf.2016.07.010)
Supplement: Supplementary file 2 [file mmc2.docx]

**Appendix B: Example usability issue data collection form**

**Participant ID : XX**

**Date : XX/XX/XX**

**Sheet no. XX**

**Task version XX**

**Sample of the *data collection form* (v.02)**

| **Usability issue**  Please describe the usability issue you have identified – ***we encourage the use of screenshots*** | **Action No.**  During which task was this issue identified? | **Heuristic category**  Under which heuristic category does this issue fall? | **Severity**  How severely do you rate this usability issue? |
| --- | --- | --- | --- |
| *Colour of selector buttons is faded giving an initial impression that they are disabled.*  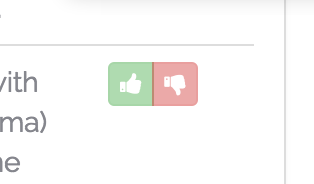 | *1.3* | *4* | *1* |
| *The text for ‘date medication increased’ doesn’t match with the graph [in terms of finding what the date is-needs hover over/ could add date to the text]*  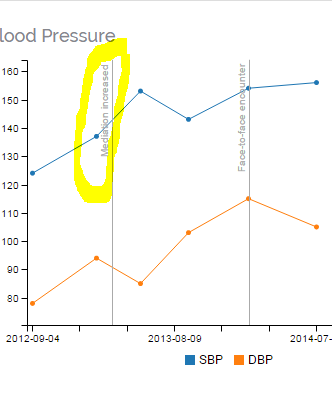 | *6.1* | *2* | *3* |
