# Supplementary material for: Interface design recommendations for computerised clinical audit and feedback: Hybrid usability evidence from a research-led system
Source: Int J Med Inform. 2016 Oct;94:191–206. doi: 10.1016/j.ijmedinf.2016.07.010 (PMC5015594; doi:10.1016/j.ijmedinf.2016.07.010)
Supplement: Supplementary file 3 [file mmc3.docx]

**Appendix C: Sample of the usability issue severity evaluation form**

| **Usability heuristic** | **Task where issue occurred^[[1]](#footnote-1)^** | **Rating of severity^[[2]](#footnote-2)^** | | | | |
| --- | --- | --- | --- | --- | --- | --- |
| **Visibility of system status** |  | **0** | **1** | **2** | **3** | **4** |
| Not clear the system status after a suggested action has been checked as completed by the user. For instance it is not clear whether the action is actually saved. | Action 1.1 |  |  |  |  |  |
| When disagreeing with a suggested action, the follow up “Why” dialog box prompting the user to justify his decision has lost context with the previous action because it covers this area of the screen. Users should know what “why?” refers to (i.e. to which action it refers to) without having to refer back to the greyed out strikethrough action. | Action 2.1 |  |  |  |  |  |
| The path used to note the current position in the system, e.g. “blood pressure > monitoring” should be made more visible. | All |  |  |  |  |  |
| It is not made immediately visible the new status of the system when a new patient has been selected from the list of patients. | Actions 3.1 and 4.1 |  |  |  |  |  |
| When you select one of the ‘improvement opportunities ‘ it is not immediately apparent that anything has changed in the patient list unless the number of patients in the list changes substantially. | Action 3.3 |  |  |  |  |  |
| When switching between NICE and QQF the new system status is not visible. | Action 8.2 |  |  |  |  |  |
| **Match between the system and the real world** |  |  |  |  |  |  |
| The (X) glyph used to represent exclusions (excluded patients) does not seem relevant. Usually this type of X symbols are used to indicate a forbidden action or exit from the current status/action. | Action 3.2 |  |  |  |  |  |
| Difficult to browse the patient list – Ordering options not helpful and visible. | Action 6.1 |  |  |  |  |  |
| Use of vague terminology in the captions of graphs – For example, What do you mean by ‘reasons we think’ who are we and what reasons? There should be more information provided regarding the captions of the different variables presented in figures. | Action 6.3 |  |  |  |  |  |
| In the individual patient graph for blood pressure readings, the Date format has the year first. However, the format dd/mm/yy would have been the most obvious option. | Action 6.3 |  |  |  |  |  |

1. For detailed description of tasks please refer to the attached “Heuristic_evaluation_tasks” document. [↑](#footnote-ref-1)
2. For a detailed description of the rating scale please refer to the attached “Rating score” document. [↑](#footnote-ref-2)
